# Impact of HPV in Oropharyngeal Cancer

**DOI:** 10.1155/2011/509036

**Published:** 2010-12-29

**Authors:** Linda Marklund, Lalle Hammarstedt

**Affiliations:** Department of Oto-Rhino-Laryngology, Head and Neck Surgery, Karolinska University Hospital, 171 76 Stockholm, Sweden

## Abstract

The incidence of oropharyngeal cancers has increased in the western world and Human Papilloma Virus (HPV) has been recognised as a risk factor in the last decades. During the same period the prevalence of HPV in oropharyngeal tumours has increased and HPV has been suggested responsible for the increase. The HPV-positive tumours are today recognized as a distinct subset of head and neck cancers with its own clinopathological and risk profile and have a significantly improved prognosis regardless of treatment strategy. This review summarizes current knowledge regarding human papillomavirus biology, oncogenic mechanisms, risk factors, and impact of treatment.

## 1. Introduction

Head and neck squamous cell carcinoma is the sixth most common cancer worldwide [1]. The incidence of head neck cancers varies widely around the world and even within populations. Oral and oropharyngeal cancer constitutes 3–5% of the malignancies in Europe, while this figure in parts of Southeast Asia and India reaches up to 40–50% [2–4]. Eighty to ninety percent of head and neck cancer cases are considered to be associated with known risk factors, such as smoking, betel nut chewing, and alcohol abuse [4, 5]. 

The prognosis for HNSCC is generally low in the more advanced stages and there has been only a modest improvement in recent years and the treatment frequently sentences the patient to life-long sequelaes such as difficulties with swallowing, dryness of the mouth, esophageal strictures, and osteoradionecrosis. Head and neck cancer is a heterogeneous group that differs greatly in tumour aggressiveness and response to treatment. The treatment is today based on tumour stage which thus leads to suboptimal outcome for some patients. The identification of predictive markers is urgent to enable optimization of treatment and reduction of sequalae for the individual patient. 

Despite a decreasing incidence of head and neck squamous cell carcinoma in general, attributed to a decrease in the prevalence of smoking [6], the incidence of oropharyngeal SCC is rising [7–12]. Human papillomavirus (HPV) has for some time been suggested to be involved in the carcinogenesis of oropharyngeal cancer. The Agency for Research on Cancer (IARC) now recognizes HPV as a risk factor for oropharyngeal cancer, and accumulating molecular and epidemiological data now show that high-risk types of HPV are responsible for a subset of oropahryngeal cancer [13–15]. HPV-positive cancers cases are now in majority in the western world and these tumours are also shown to have better outcome than the HPV-negative patients. However, the natural history and the tumour development biology of HPV-infection in head and neck tumours are not yet fully understood and the best management needs further investigation and clinical trials in order to achieve the best clinical outcome for the patients with HPV-positive head and neck tumours.

## 2. Human Papilloma Virus (HPV) and Tumour Biology

HPV was first identified in 1949 [16] and today over 100 different HPV types have been characterized [17]. HPVs are small, circular double stranded DNA viruses with a genome that consists approximately 8000 base pairs. HPV infection is highly restricted to basal cells in the mucosal or epithelial layers. Replication occurs within the nucleus of the infected cell and is dependent on S-phase entry since it requires the DNA machinery [18]. The HPV subtypes are divided into high-risk and low-risk HPV regarding their malignant potential. Approximately 15 high-risk subtypes are known but only HPV subtypes 16, 18, 31, 33, and 35 have been identified playing a role in the development of oropharyngeal head and neck cancer. HPV 16 is the far most common type detected in oropharyngeal cancer accounting for 90–95% of the HPV positive tumours [19]. 

High-risk HPVs produce 2 oncoproteins, E6 and E7, which are necessary for viral replication through their proliferation stimulating activity and play a key role in malignant transformation and maintenance. The E6 oncoprotein binds and induces the degradation of the p53 tumour suppressor protein via an ubiqutin-mediated process disrupting the p53 pathway which leads to uncontrolled cell cycle progression [20, 21]. The HPV E7 protein binds and degrades the retinoblastoma protein (pRb), preventing it from inhibiting the transcription factor E2F resulting in loss of cell cycle control. Furthermore, the functional inactivation of Rb results in upregulation of the p16-protein. P16 is encoded by the CDKN2A tumour suppressor gene and regulates the activity of CyklinD-CDK4/6 complexes that phosphorylate Rb leading to release of the transcription factor E2F which initiates cell cycle progression. The bound Rb-E2F protein acts as a negative regulator by inhibiting transcription CDKN2A, and therefore the functional inactivation of Rb by E7 thereby the transcriptional inhibition of the p16 gene is lost. HPV-positive tumours are consequently characterised by high expression of high levels of p16 [22]. p16 protein can be detected by technically simple immunohistochemistry, and since several studies have shown a very high correlation (>90%) to HPV-positively in oropharyngeal tumours, it has been suggested as a clinically useful sorrogatemarker [23, 24].

In head and neck cancer caused by the traditional risk factors, tobacco and alcohol, p53 is commonly mutated [25, 26] and 9p21-22 is lost early in carcinogenesis resulting in the loss of the tumour suppressing gene p16 [27]. In contrast, HPV-positive head and neck tumours have decreased expression of wild-type p53 due to the inactivation and degradation by the E6 oncoprotein. Furthermore, in a study by Westra et al. in 2008 [28] it was shown that HPV 16 and mutated 53 may coexist in a subset of head and neck squamous cell carcinoma but HPV 16 and disruptive p53 mutations seemed to be nonoverlapping events. An inverse relationship between HPV-16 infection and disrupted p53 gene mutations in head and neck carcinomas was suggested, and thus HPV positive head and neck tumours represent a distinct molecular phenotype with a unique mechanism of tumorigenesis independent of the mutagenic effect of tobacco and alcohol. 

## 3. Risk Factors for HPV

In contrast to the HPV-negative cancers in the head neck region the vast majority of HPV-positive cancers lack association with the traditional risk factors, tobacco and alcohol [29]. Epidemiological studies on HPV-associated cervical cancer have clearly demonstrated that HPVs are transmitted by sexual contact [30] and today there are several studies suggesting that also HPV-positive head and neck tumours are sexually transmitted. It is assumed that HPV infection precedes the development of HPV positive head and neck cancers, and the presence of high-risk HPV infection on the oral mucosa and seropositivity increase the risk of development of head and neck cancers [13, 14, 31, 32]. Therefore risk factors for HPV oral infection are likely to be risk factors for HPV-positve head neck tumours. Oral HPV infections are rare in newborns of HPV-infected mothers and in children prior to sexual activity; infections increase after onset of sexual activity. In addition, an increased risk for tonsillar cancer among women with cervical lesions and a higher rate of tonsillar and tongue cancers among husbands of women with cervical dysplasia or cancer have been identified [33]. The risk of HPV-positive head and neck tumours has been associated with sexual behaviour including increasing numbers of both vaginal and oral sex partners, young age at first intercourse, and history of genital warts [34–37]. Also other life style factors like poor oral hygiene and marijuana use have been discussed and the most recent report found that the risk of developing an HPV-16 positive head and neck cancer increased with increased marijuana use but no correlation to oral hygiene was found [37]. Whether tobacco and alcohol increase the risk is not clear where some studies found no association to development of HPV-positive head neck cancers [13, 37] while others have found that thier use increase the risk [38]. Patients with the Fanconi anaemia have increased risk of HPV-mediated tumourigenesis [13] and some data indicate that HIV-patients have increased rates of HPV-related disease in the oral cavity despite antiretroviral therapy [39, 40].

## 4. Epidemiology

As for other head and neck cancers, the incidence of orophayrngeal cancer rates varies widely around the world and even within population significant differences have been observed. The black population in the United States, for instance, tends to have higher incidence rates than whites and hispanic populations all over the country. The SEER, Surveillance Epidemiology and End Results, covers approximately 14% of the US population and the age-standardized rates of tonsillar cancer in whites were 1.4 for men and 0.4 for women in 1993–1997. For blacks the rates were 2.9 and 0.6, per 100 000 person-years, respectively. 

By contrast, in China, the rates of tonsillar cancer are generally low, for instance, in Beijing where the rates were 0.1 for men and 0.0 for women, respectively. Interestingly, in Hong Kong and in Taiwan, places with great western influence, the rates were 6 to 12 times higher than in Beijing. In India, with high rates of oral cancer, the rates of tonsillar cancer were between 0.8 and 2.8 in men and 0.2 and 0.5 in women, respectively.

 In most countries the rates tend to be higher in males than in females with a ratio from 2 : 1 to 5 : 1. However, in the Philippines and in Vietnam women had higher incidence rates than men. Interestingly, this was true also for the Philippine population in California.

Also in Europe, the incidence rates show great variations with intranational variability in some countries. The highest rates were seen in parts of France, in Somme, where the rates were as high as 6.4 for men and 0.8 for women [41].

 In several western countries, the incidence of oropharyngeal cancer has increased greatly in the last decades [7–9, 42–44] and the incidence has increased most in men. At the same time, the prevalence of HPV in those tumours has increased in a similar way indicating that HPV in fact is responsible for this increase [11, 42, 45]. HPV has been found in 45–95% of the oropharyngeal tumours [11, 42, 46] and the prevalence of HPV 16 has been quite homogenous around the world [47] in contrast to cervical cancer, where the prevalence of different types of HPV varies around the world [48].

## 5. HPV in Head-Neck Cancers

Apart from having a different epidemiology and aetiology, the HPV-positive oropharyngeal cancers also constitute a distinct subgroup clinopathologically. HPV-positive tumours are usually poorly differentiated and nonkeratinizing and have a basaloid appearance in contrast to the HPV-negative that is more moderately differentiated and keratinizing [49, 50]. HPV-positive tumours also demonstrate significantly lower levels of chromosomal mutations than the HPV-negative tumours [51, 52]. Furthermore, patients with HPV-positive oropharyngeal cancers in general, especially tonsillar cancers, tend to be younger at time of diagnosis [42, 53], possibly with the exception of base of tongue cancers where no age difference could be found between the HPV-positive and HPV-negative cancer patients [10]. The majority of the patients have no prior history of tobacco and/or high alcohol consumption and have generally a better performance status compared to the HPV-negative patients [37, 54]. Moreover, HPV-postive tumours often present at a higher stage with a small T-size (T1-T2) [55] but frequently there is a large, often cystic, nodal involvement (N+) [56, 57], thus the HPV-positive tumours are often diagnosed in clinical advanced stages, that is, Stages III-IV [15]. 

## 6. Clinical Implications/Effect on Prognosis

The prognosis for HPV-positive oropharyngeal cancer patients is better than that for patients with HPV negative tumours independent of nodal status, age, stage, tumour differentiation, or gender [1, 15, 58–60]. Several studies have shown 80–95% 2-3 years overall survival rate for the HPV-positive patients compared to 57–62% for the HPV-negative subgroup of oropharyngeal tumours [60–62]. 

While it is still unclear whether tobacco is a risk factor for HPV-induced oropharyngeal tumors, it seems clear that smoking has a negative impact on relapse and survival for the HPV-positive tumours [63]. The risk of death and cancer relapse significantly increased by 1% for each additional pack year of tobacco smoking [61], indicating that the biological behaviour of an HPV-positive tumour may be altered by tobacco use. 

Treatment of head and neck tumours today is often standardized and based on tumour stage despite the knowledge of the heterogeneity regarding tumour aggressiveness and response to treatment of the tumours varies greatly. Treatment of patients with advanced disease often includes both oncological and surgical treatment as both carry acute side effects and lifelong sequelae. The surgical trend in the last years, especially regarding neck dissection, has turned from a very radical operation towards more organ preserving selective/modified neck dissections when possible, reducing the morbidity. In contrast, the oncological treatment has turned in the opposite direction including the development of altered fractionation radiotherapy, integration of chemotherapy with radiotherapy, incorporation of intensity-modulated radiotherapy, and the introduction of targeted biological therapy. The combined modality treatment and the altered/intensified fractionation have improved outcome for head neck cancer patients in general [64, 65], but the morbidity has also significantly increased [66–68]. However, today there is no absolute consensus about patient selection for altered fractionation regimens, type of chemo-radiotherapy association, radiation, or chemotherapy dose schedule. Among older patients with advanced disease, age over 70 years, the compliance to treatment is low due to significant comorbidity and poor performance [68]; thus this intense therapy is probably not suitable for this group of patients. 

Patients with HPV-positive tumours in the oropharynx show an improved survival regardless of treatment strategy. Superior outcome for HPV-positive tumours has been shown for surgery [69], convectional and modified fractionated radiotherapy [55], induction chemotherapy [60], concurrent chemotherapy [61] and induction chemotherapy plus concurrent chemotherapy [60]. Thus, regarding patients with HPV-positive tumours in oropharynx the debate today is whether the intense therapy is too aggressive in this group of patients since they show a superior survival regardless of treatment strategies. 

Strengthening this theory, a recent published study by Ang et al. [61] showed no significant difference in overall survival between a concomitant boost accelerated fraction regimen of radiotherapy and a standard fractionation regimen when combined with concurrent high-dose cisplatin for HPV-positive patients. 

The reason for the better response to treatment for patients with HPV positive tumours is not known. There are studies that have found an inverse relationship between tumour HPV status and presence of p53 mutations in head and neck cancer [50, 70]. The improved response to oncological treatment observed for patients with HPV-positive tumours could therefore be explained by the presence of an intact p53-mediated apoptotic response in HPV-positive tumours. Another possibility is immunological factors related to HPV infection [71]. 

Today, there is a subgroup of the HPV-positive oropharyngeal cancers that have worse clinical outcome, that do not respond as well to given treatment, and have a higher rate of relapses and worse survival than the majority of the tumours in this group. A question for the future is how to separate the HPV-positive oropharyngeal tumours in this relative small group. Several potential clinical markers have been suggested, p16 [23, 24, 55, 72], Cyklin D1, [73], EGFR [74–76], p53 [28], and p21 [73], and maybe a combination of these and other not yet known markers may provide additional prognostic information and thus guide us to select the right patients for the right combination of treatment. 

Based on the profound impact of HPV on the response to treatment for patients with oropharyngeal tumours, HPV-status should be included to standard pathological reporting in clinic and the planning of treatment strategies for this group of patients. Future clinical trials comprising patients with oropharyngeal tumours should include HPV status as a stratification factor in order to identify the least morbid treatment to cure this group of patients.

## 7. Concluding Remarks

HPV-positive oropharyngeal cancer is recognized as a distinct subset of head and neck squamous cell carcinoma with a favourable outcome. HPV status is a profound prognostic factor for overall and progression-free survival, treatment response, and tumour control. The superior outcome is independent of nodal status, age, stage, tumour differentiation, or gender and regardless of treatment strategy. The explanation for this difference is not clearly understood today and is probably a combination of several factors, patient-related (younger age, less exposed to tobacco and alcohol, less co morbidity, etc.) as well as tumour-related factors (presence of p53-mediated apoptosis). 

HPV-positive oropharyngeal tumours represent a distinct clinopathological profile where there still exist many questions to be answered regarding tumour biology and how the disease develops (tumouregenesis). 

In future, clinical trials on oropharyngeal tumours need to take HPV-status in consideration. There is a possibility that less intense treatment strategies with lower rate of acute and long-term side effects do not compromise the survival outcome for this group of patients. Since patients with HPV-positive oropharyngeal cancers often are younger and in good health, few severe life-long complications are very important considerations. We need further knowledge about the tumour biology and identification of additional clinical useful markers to combine with HPV-status for appropriate risk stratification in future clinical trials in order to optimize the treatment for each individual patient in the future. 
